# Supplementary material for: TWEAK/Fn14 signalling driven super-enhancer reprogramming promotes pro-metastatic metabolic rewiring in triple-negative breast cancer
Source: Nat Commun. 2024 Jul 5;15:5638. doi: 10.1038/s41467-024-50071-z (PMC11224303; doi:10.1038/s41467-024-50071-z)
Supplement: Supplementary file 1 — Supplementary Information [file 41467_2024_50071_MOESM1_ESM.pdf]

Supplementary material

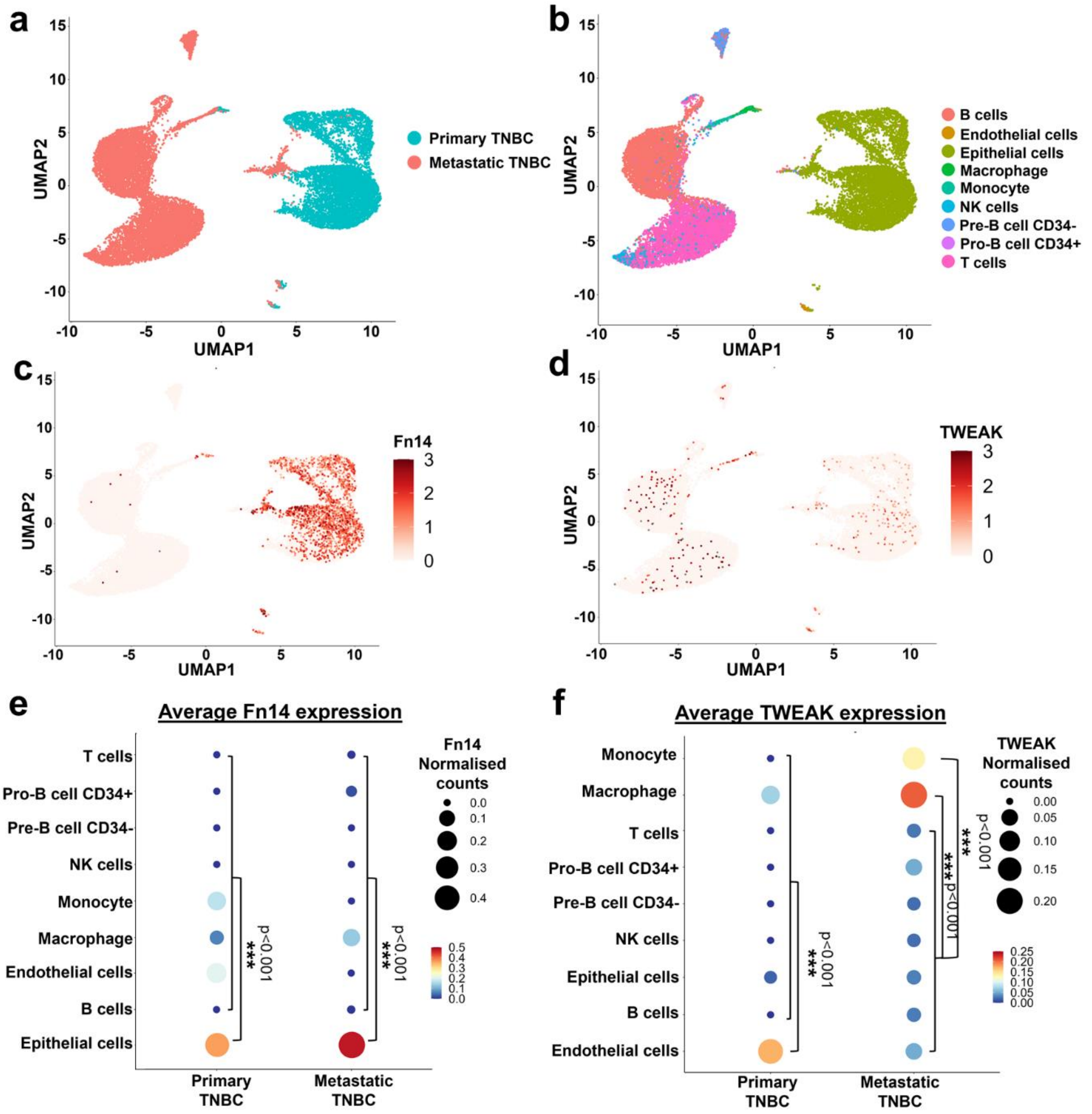

Supplementary Figure 1. scRNA-seq analyses of primary TNBC and its lymph node metastases reveal that *Fn14* is mainly expressed in breast cancer epithelial cells while *TWEAK* is mainly expressed in endothelial cells and macrophages. UMAP overview of cells from primary TNBC tumour and its matched lymph nodes with metastasis detected, analysed by scRNA-seq, acquired from (1) depicting (a) the origin of each cell, (b) the cell type, and the expression of (c) *Fn14* and (d) *TWEAK* of each cell. Plot depicting the average (e) *Fn14* and (f) *TWEAK* expression of single cells in the primary TNBC tumour and lymph node metastases. Two-sided t-test was used for statistical analysis comparing epithelial cells or endothelial cells versus all other cell types, monocytes versus all other cell types except macrophages and macrophages versus all other cell types except monocytes. *P < 0.05; **P < 0.01; ***P < 0.001.

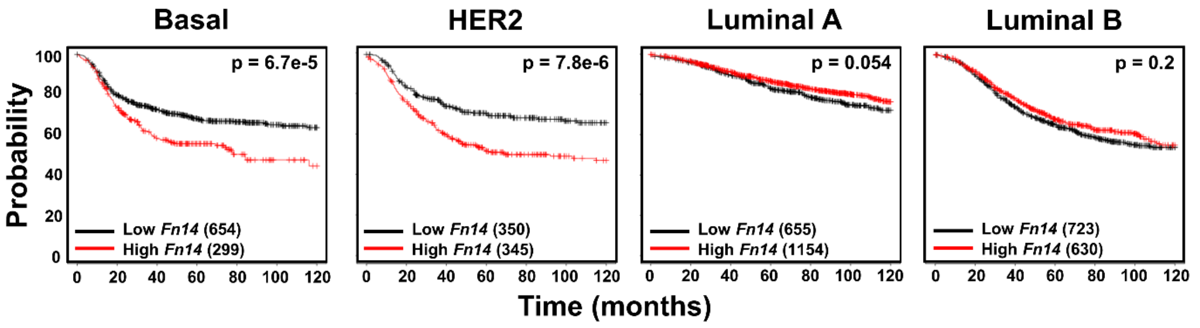

Supplementary Figure 2. High Fn14 expression confers worse survival in Basal-like and HER2 patients. Kaplan Meier plot depicting the relapse-free survival of *Fn14* high and low Basal, HER2, Luminal A and Luminal B breast cancer patients.

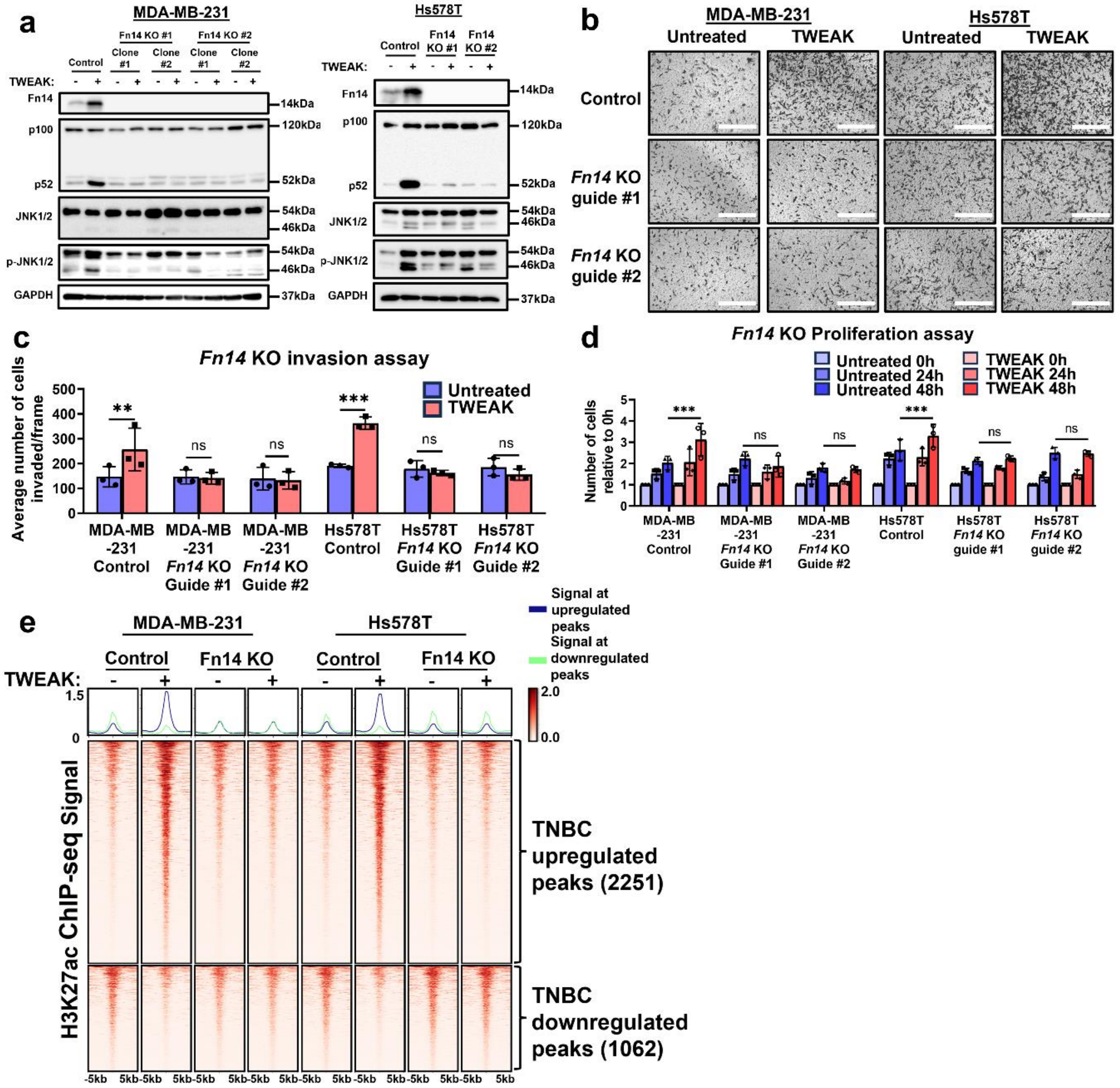

Supplementary Figure 3. Fn14 KO abolishes TWEAK/Fn14-driven intracellular signalling pathway activation, invasion, cell proliferation and enhancer changes in TNBC cell lines. (a) Fn14, NF-κB and MAPK pathway signalling regulators analysed by western blotting in control and Fn14 KO MDA-MB-231 and Hs578T cells, treated with and without TWEAK. (b) Transwell invasion assay was performed in control and Fn14 KO MDA-MB-231 and Hs578T cells with and without TWEAK treatment. Representative images from n=3 biological replicates are shown. Scale bars: 400μm. (c) Plot depicts the average number of invaded cells/frame (mean ± s.d) from n=3 biological replicates, across four fields per replicate. (d) Proliferation assay plot depicts the average number of cells counted relative to 0h (mean ± s.d) in control and Fn14 KO MDA-MB-231 and Hs578T cells with and without TWEAK from n=3 biological replicates. (e) H3K27ac ChIP-seq signals of control and Fn14 KO MDA-MB-231 and Hs578T cells, treated with and without TWEAK at differentially regulated enhancer sites in TNBC cell lines. The western blot samples derive from the same experiment but different gels for NFKB2 and GAPDH, another for Fn14 and another for p-JNK1/2 and JNK1/2 were processed in parallel. Experiments involving cell lines were performed 3 times independently, each time on different days. Two-sided two-way ANOVA was used for statistical analysis in *in vitro* proliferation and invasion assay. *P < 0.05; **P < 0.01; ***P < 0.001. Source data are provided as a Source Data file.

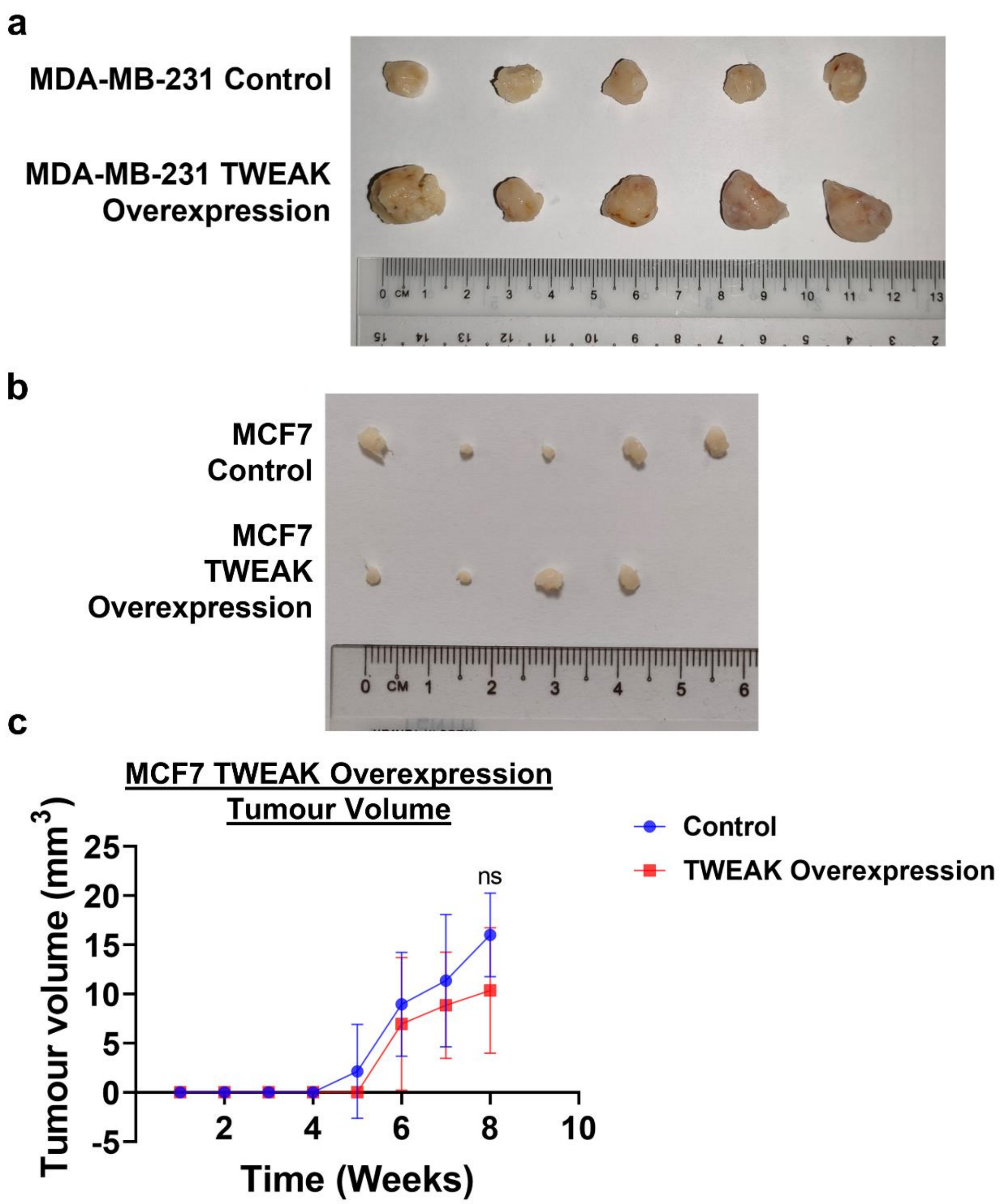

Supplementary Figure 4. Persistent Fn14 activation promotes tumour growth in TNBC but not ER-positive breast cancer cells. Images of tumours extracted from mice 8 weeks after being injected with (a) luciferase expressing MDA-MB-231 cells (n=5 biological replicates) and (b) MCF7 cells (n=5 biological replicates) harbouring the overexpression control or TWEAK overexpression. There was no tumour growth in the 5th MCF7 TWEAK Overexpression mouse. (c) Tumour growth plot depicts the weekly average tumour volume (mean ± s.d) from mice injected with MCF7 cells overexpressing luciferase and overexpression control or TWEAK. Two-sided t-test was used for statistical analysis. Source data are provided as a Source Data file.

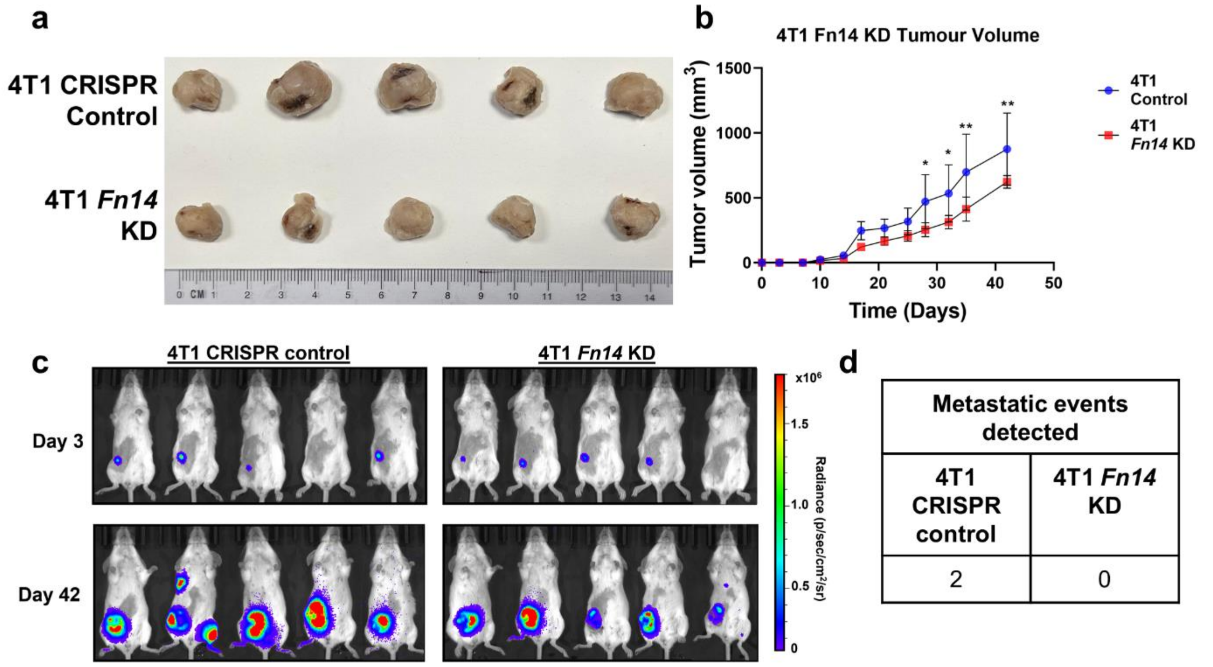

Supplementary Figure 5. *Fn14* KD reduces tumour growth and metastasis in immune competent BALB/cN mice. (a) Images of tumours extracted from mice 42 days after being injected with luciferase overexpressing 4T1 cells harbouring either CRISPR control or *Fn14* KD (n=5 biological replicates). (b) Tumour growth plot depicts the weekly average tumour volume (mean ± s.d) from mice injected with 4T1 cells overexpressing luciferase and harbouring either CRISPR control or *Fn14* KD. (c) IVIS tracking of mice injected with 4T1 cells overexpressing luciferase and harbouring either CRISPR control or *Fn14* KD. Representative bioluminescent images of the animals were taken at days 3 and 42 after orthotopic xenograft. (d) Plot depicts the number of metastatic events observed in animals after 42 days in n=5 biological replicates. Data presented as binomial. Metastasis detected in control mice #2 and #4. Two-sided t-test was used for statistical analysis in tumour volume growth assay. *P < 0.05; **P < 0.01; ***P < 0.001. Source data are provided as a Source Data file.

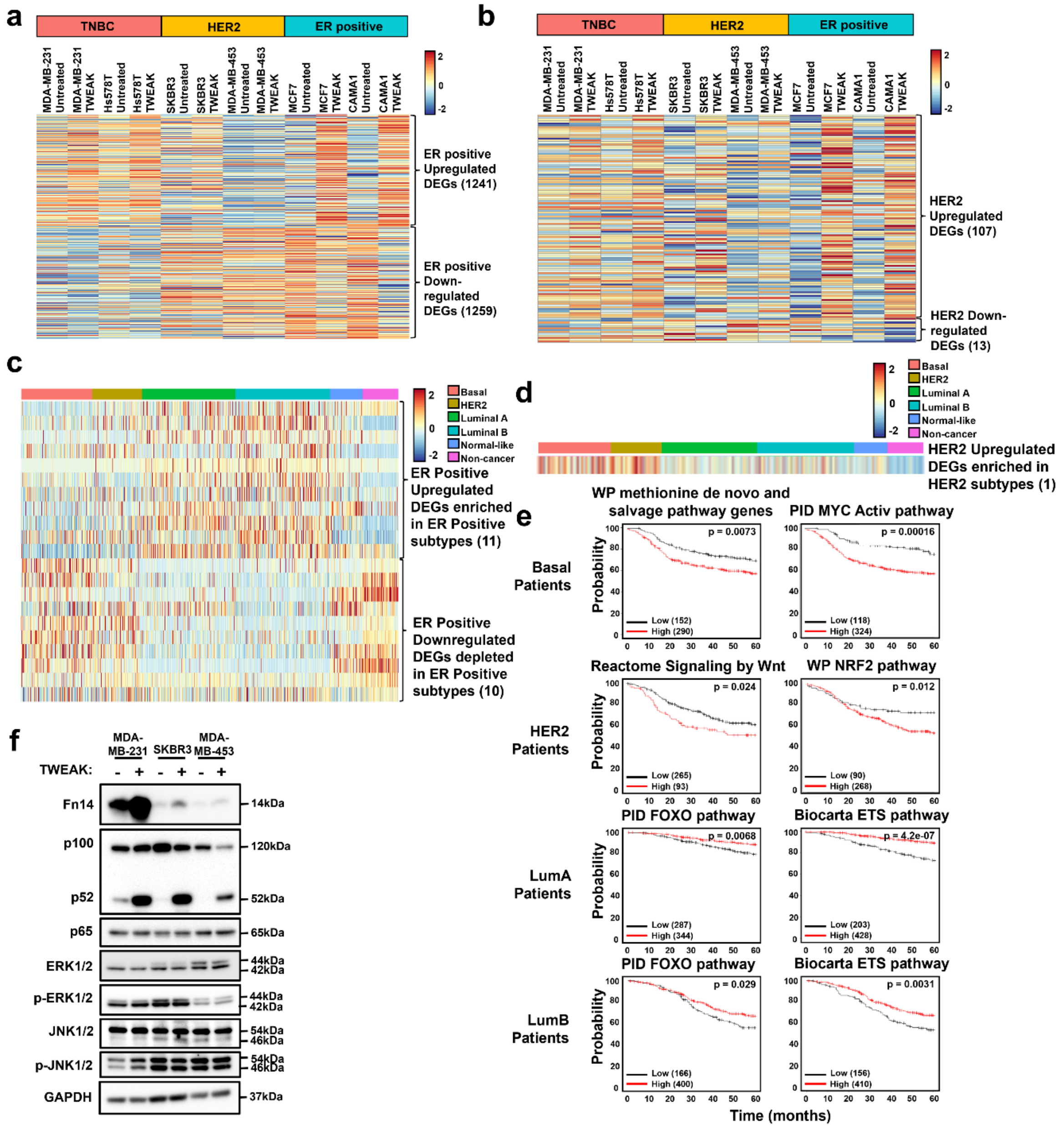

Supplementary Figure 6. TWEAK/Fn14-induced transcriptional signatures in HER2 and ER-positive breast cancer cell lines are not enriched in their respective tumour subtypes. Heatmap depicting the average expression of TWEAK/Fn14 differentially regulated (a) ER-positive genes and (b) HER2 genes in MDA-MB-231, Hs578T, SKBR3, MDA-MB-453, MCF7 and CAMA1 cell lines with and without TWEAK. Data shown represent n=3 biological replicates. Heatmap depicting the expression of TWEAK/Fn14 upregulated and downregulated genes in (c) ER-positive cell lines and (d) HER2 cell lines that were respectively enriched and depleted in patients of their respective TCGA BRCA subtype. (e) Kaplan Meier plot depicting the relapse-free survival of Basal, HER2, Luminal A and Luminal B breast cancer patients based on the average expression of genes associated with TWEAK-activated subtype-specific enriched pathways in Fig. 2d. (f) NF-κB and MAPK pathway signalling regulators analysed by western blotting in MDA-MB-231, SKBR3 and MDA-MB-453 cells, treated with and without TWEAK. MDA-MB-231 is added as a reference for comparison against Figure 2e. The western blot samples derive from the same experiment but different gels for Fn14 and p65, another for NFKB2 and GAPDH, another for p-JNK1/2 and JNK1/2, and another for p-ERK1/2 and ERK1/2 were processed in parallel. Source data are provided as a Source Date file.

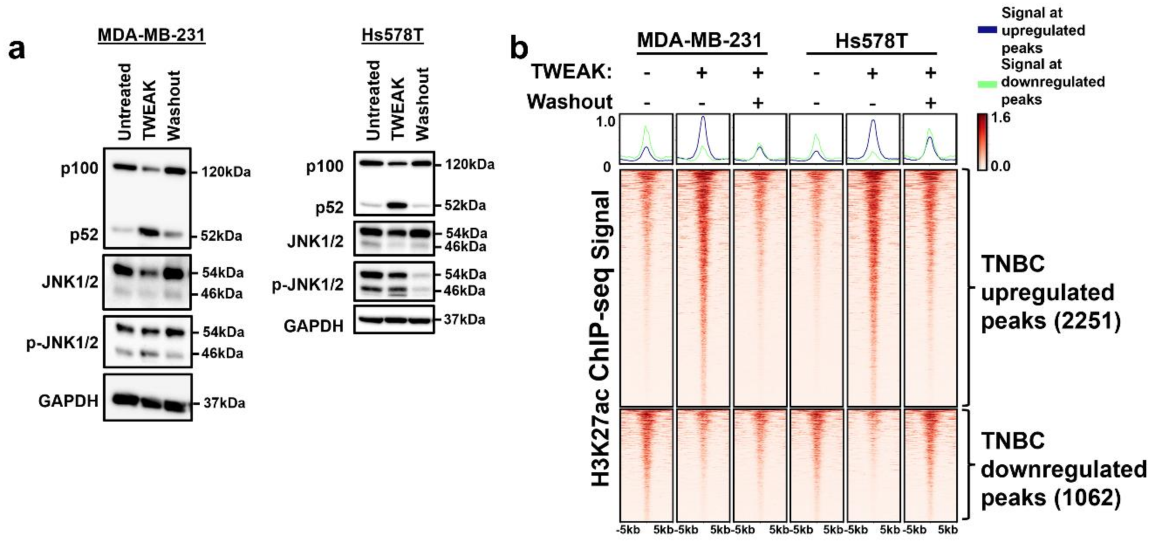

Supplementary Figure 7. Wash off ameliorates TWEAK-driven non-canonical NF-κB pathway, JNK pathway and enhancer activation in TNBC. (a) NF-κB and JNK pathway signalling regulators analysed by western blotting in untreated, TWEAK-treated and TWEAK-treated with 96h wash-off MDA-MB-231 and Hs578T cells. (b) H3K27ac ChIP-seq signals of untreated, TWEAK-treated and TWEAK-treated with 96h wash-off MDA-MB-231 and Hs578T cells at TWEAK/Fn14-driven differential TNBC enhancer sites. The western blot samples derive from the same experiment but different gels for NFKB2 and GAPDH and another for p-JNK1/2 and JNK1/2 were processed in parallel. Source data are provided as a Source Data file.

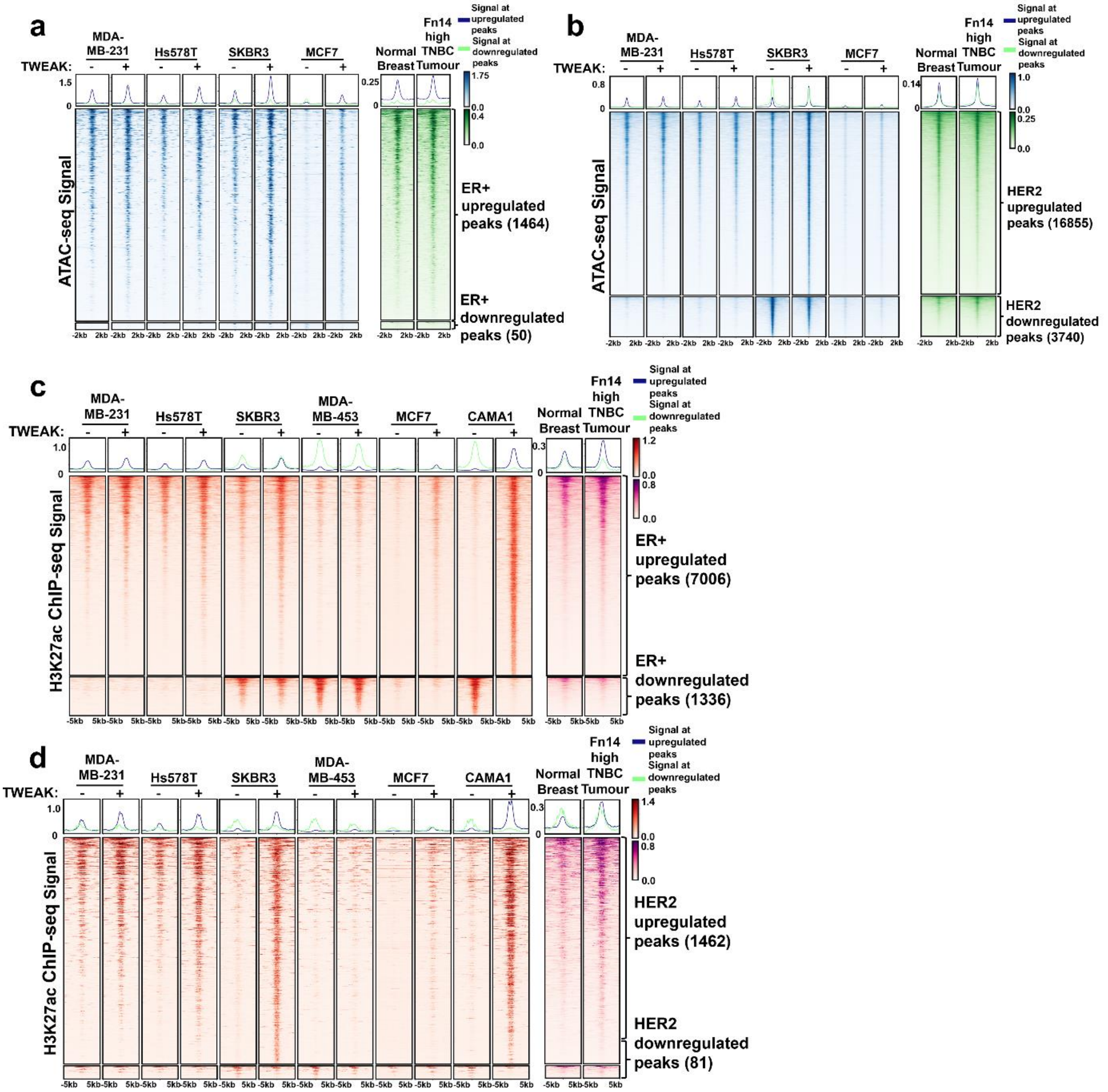

Supplementary Figure 8. TWEAK/Fn14-driven chromatin accessibility and enhancer dynamics in HER2 and ER-positive breast cancer cell lines are not subtype specific. ATAC-seq signals of MDA-MB-231, Hs578T, SKBR3 and MCF7 with and without TWEAK, as well as the merged signal of Fn14 high TNBC patient tumours and their matched normal samples (2086, 8370, 2963, 6122, 8850 and 5929) at differentially regulated chromatin accessible sites in (a) MCF7 and (b) SKBR3 cells following TWEAK treatment. H3K27ac ChIP-seq signals of MDA-MB-231, Hs578T, SKBR3, MDA-MB-453, MCF7 and CAMA1 with and without TWEAK, as well as the merged signal of Fn14 high TNBC patient tumours and their matched normal samples at differentially regulated enhancer sites in the (c) ER-positive and (d) HER2 cell lines.

a

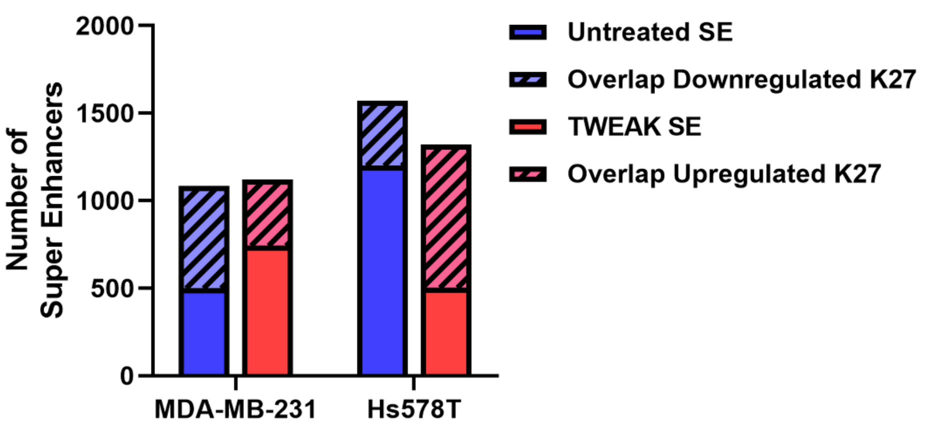

b

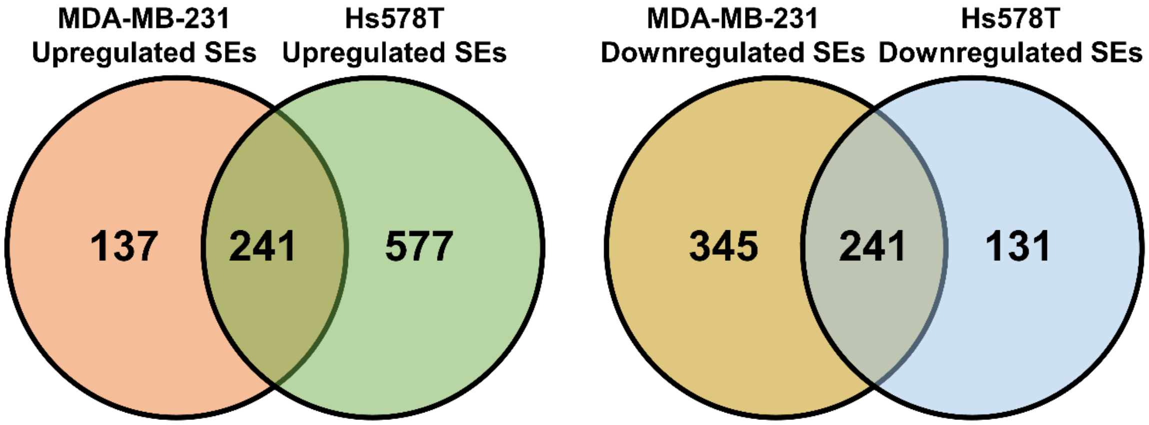

Supplementary Figure 9. TWEAK/Fn14 signalling is highly involved in TNBC SE regulation. (a) Bar plot depicting the number of SEs called in MDA-MB-231 and Hs578T cells with and without TWEAK treatment, as well as the number of SEs that overlap up- and down-regulated TNBC H3K27ac ChIP-seq peaks. (b) Venn diagrams depicting the number of common and cell line specific TWEAK/Fn14 regulated SEs between MDA-MB-231 and Hs578T cells.

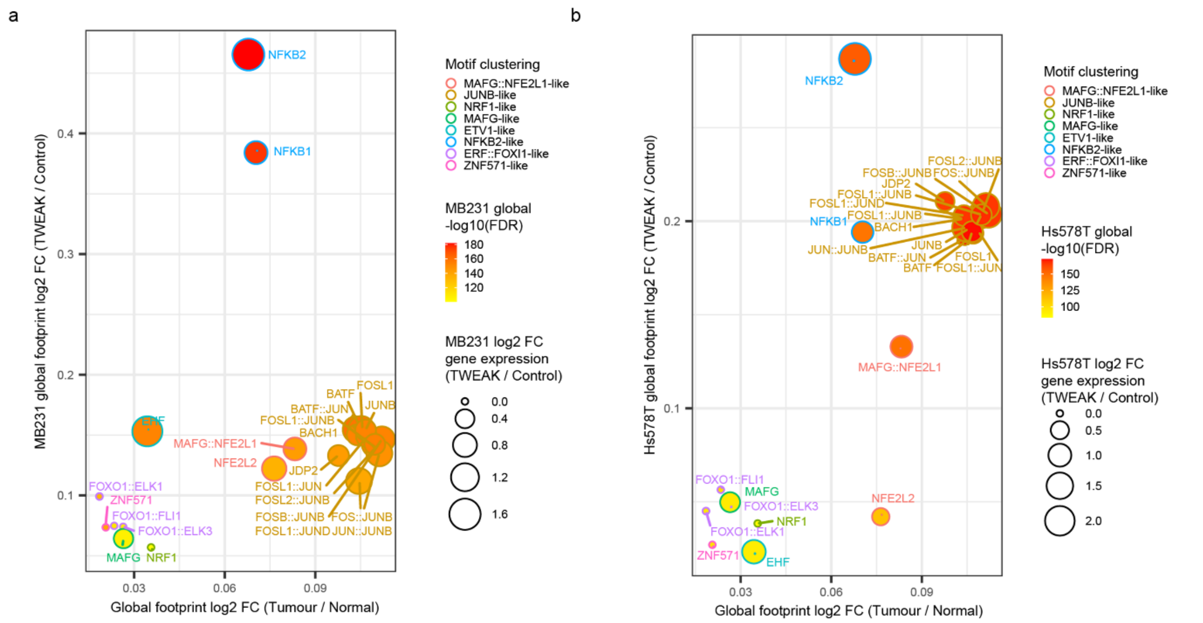

Supplementary Figure 10. Global TF DNA binding dynamics in TWEAK stimulated MDA-MB-231, Hs578T and Fn14-high TNBC patient tumours. (a) MDA-MB-231 and (b) Hs578T global log2 fold changes for footprints and gene expression are plotted with Fn14 high TNBC patient tumour global footprint log2 fold changes. TF binding changes exhibiting p-values in the upper half of the distribution are selected and filtered for consistent global footprint and gene expression dynamics across cell lines and tumours. Motifs are clustered based on similarity via TOBIAS.

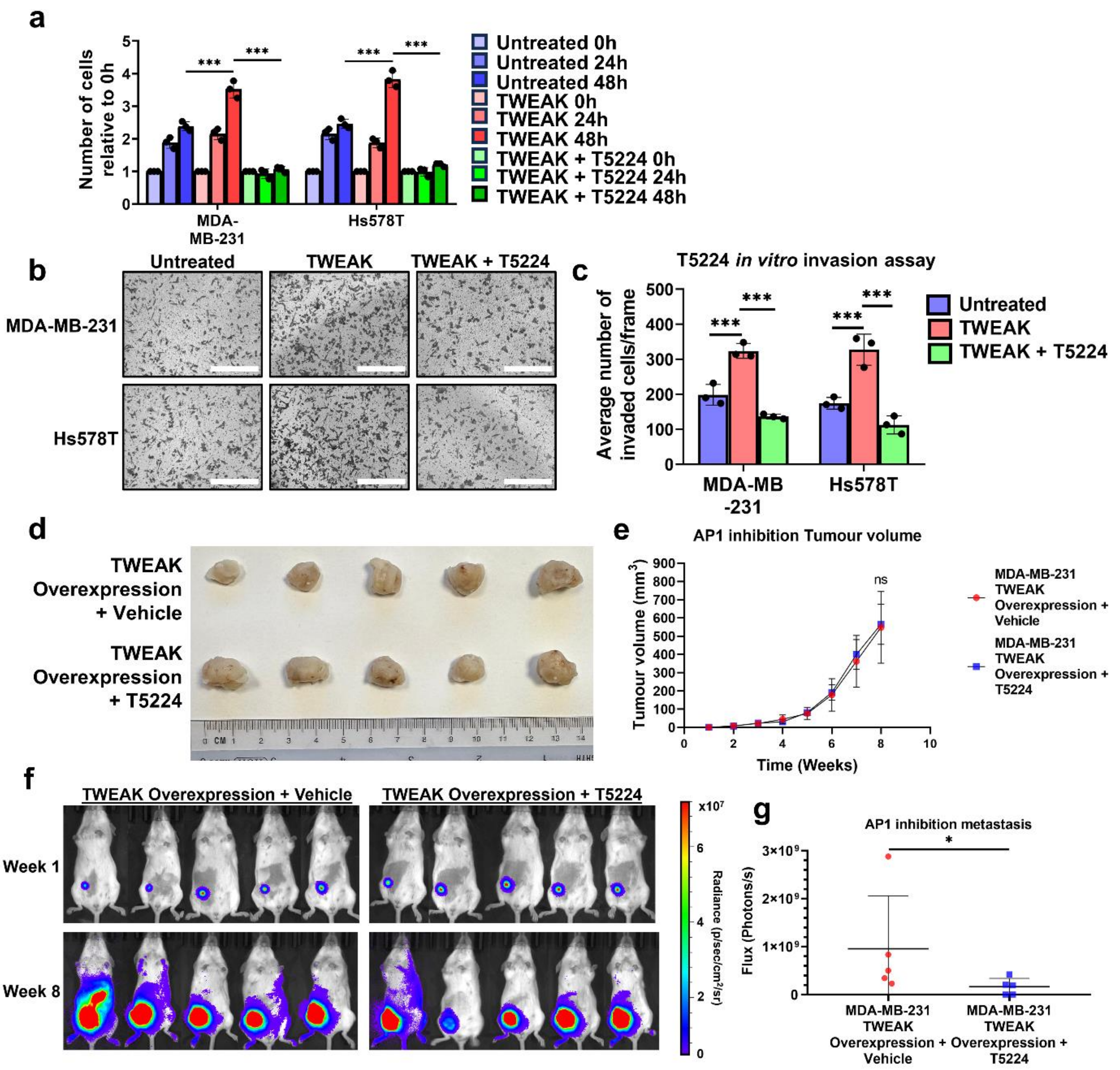

Supplementary Figure 11. AP1 inhibition through T5224 abolishes TWEAK/Fn14 driven proliferation and invasion *in vitro*, as well as metastasis *in vivo*. (a) Proliferation assay plot depicts the average number of cells counted relative to 0h (mean ± s.d) in untreated, TWEAK-treated and TWEAK + 10μM T5224 treated MDA-MB-231, Hs578T, n=3 biological replicates. (b) Transwell invasion assay was performed in untreated, TWEAK-treated and TWEAK + 10μM T5224 treated MDA-MB-231, Hs578T. Representative images from n=3 biological replicates are shown. Scale bars: 400μm. (c) Plot depicts the average number of invaded cells/frame (mean ± s.d) from n=3 biological replicates, across four fields per replicate. (d) Images of tumours extracted from mice 8 weeks after being injected with luciferase and TWEAK overexpressing MDA-MB-231 cells and treated with vehicle or 10μM T5224 (n=5 biological replicates). (e) Tumour growth plot depicts the weekly average tumour volume (mean ± s.d) from mice injected with MDA-MB-231 cells overexpressing luciferase and TWEAK, treated with vehicle or 150mg/kg T5224. (f) IVIS tracking of mice injected with MDA-MB-231 cells overexpressing luciferase and TWEAK, treated with vehicle or 150mg/kg T5224. Representative bioluminescent images of the animals were taken at 1 and 8 weeks after orthotopic xenograft. (g) Plot depicts total flux at the metastatic sites of each animal after 8

weeks in n=5 biological replicates. Experiments involving cell lines were performed 3 times independently, each time on different days. Two-sided two-way ANOVA was used for statistical analysis in *in vitro* proliferation and invasion assay. Two-sided t-test was used for statistical analysis in *in vivo* assays. *P < 0.05; **P < 0.01; ***P < 0.001. Source data are provided as a Source Data file.

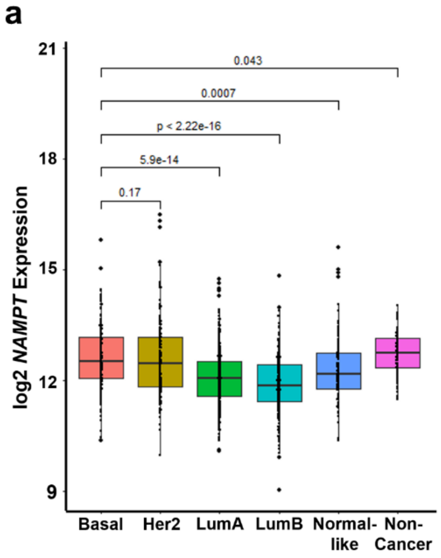

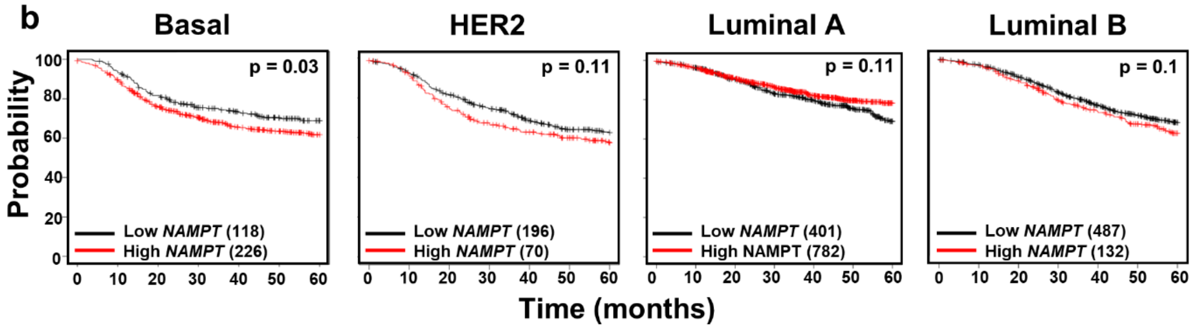

Supplementary Figure 12. NAMPT is overexpressed in ER-negative breast tumours and its overexpression confers worse survival selectively in basal-like breast cancer patients. (a) Relative *NAMPT* gene expression levels in Basal-like, HER2, Luminal A, Luminal B, Normal-like and non-cancer patient samples from the TCGA BRCA RNA-seq dataset. (b) Kaplan Meier plot depicting the relapse-free survival of *NAMPT* high and low Basal-like, HER2, Luminal A and Luminal B breast cancer patients. Two-sided Wilcoxon signed-rank test was used for differential gene expression.

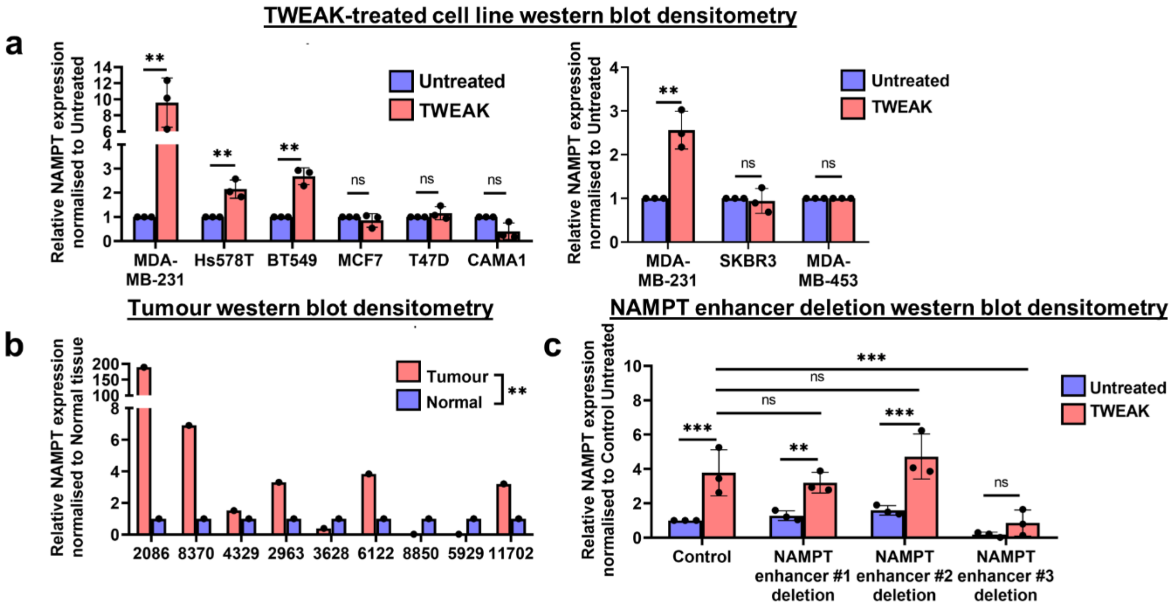

Supplementary Figure 13. Western blot densitometries. Relative NAMPT expression to GAPDH, (a) normalised to each cell line's untreated sample in Figure. 6a, (b) normalised to each tumour sample's matched normal tissue in Figure 6b and (c) normalised to the control untreated sample in Figure 6d. Two-sided t-test was used for statistical analysis, where $*P < 0.05$; $**P < 0.01$; $***P < 0.001$. Source data are provided as a Source Data file.

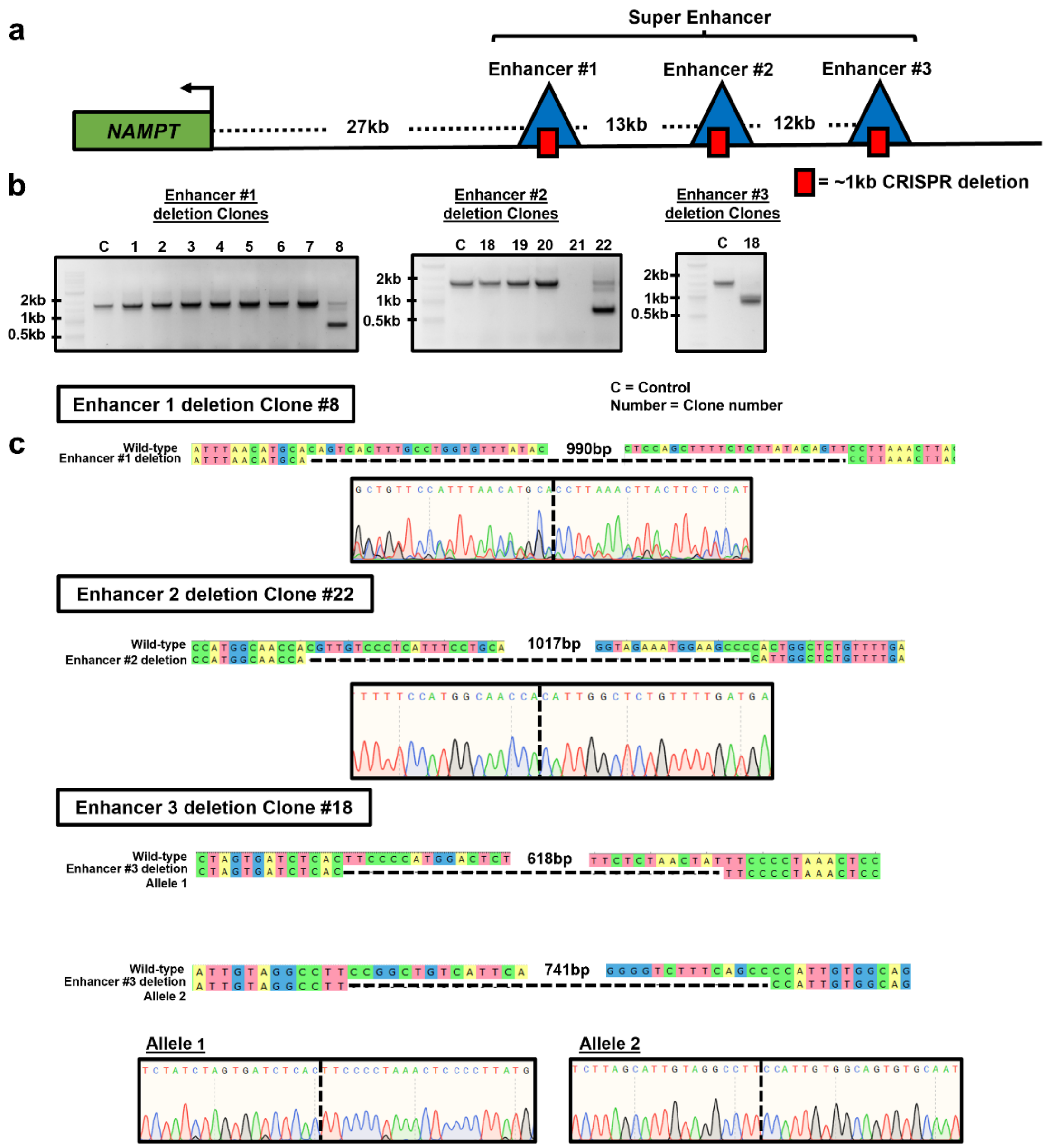

Supplementary Figure 14. *NAMPT* enhancer CRISPR deletion strategy and genotyping of CRISPR-edited clones. (a) Diagram depicting the *NAMPT* locus and the locations where the 1kb CRISPR/Cas9 deletions were made. (b) Gel electrophoresis images of MDA-MB-231 clones harbouring successful deletions in *NAMPT* enhancer #1, #2 and #3. (c) Sanger sequencing tracks showing the regions in *NAMPT* enhancer #1, #2 and #3 that were successfully deleted in MDA-MB-231 clones. Source data are provided as a Source Data file.

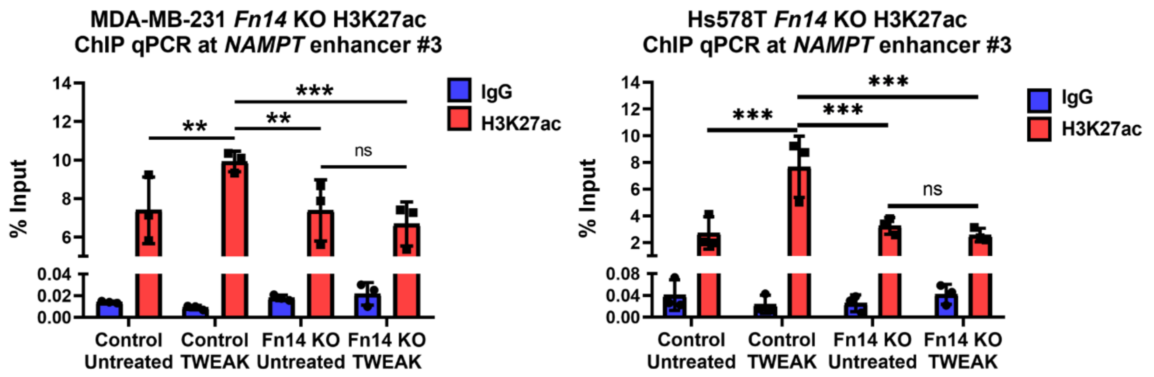

Supplementary Figure 15. Fn14 KO abolishes TWEAK/Fn14-driven *NAMPT* enhancer #3 regulation. Plot depicting the IgG and H3K27ac ChIP qPCR %input at *NAMPT* enhancer #3 in control and Fn14 KO MDA-MB-231 and Hs578T cells with and without TWEAK treatment (mean ± s.d). Data shown represent n=3 biological replicates. Two-sided two-way ANOVA was used for statistical analysis where *P < 0.05; **P < 0.01; ***P < 0.001. Source data are provided as a Source Data file.

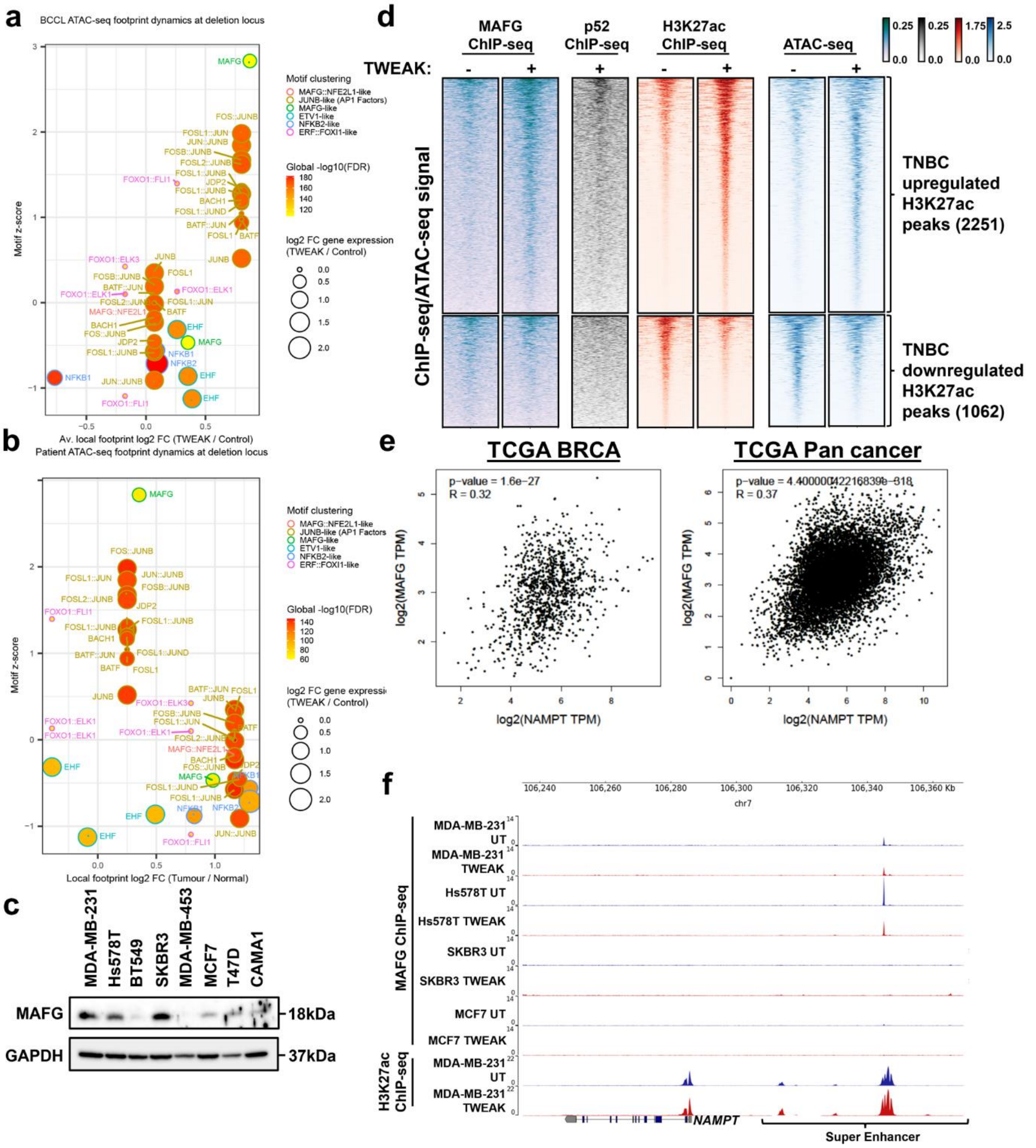

Supplementary Figure 16. MAFG binds to *NAMPT* enhancer #3 in TNBC and is positively correlated with NAMPT expression. (a) Average local log2 fold changes for MDA-MB-231 and Hs578T footprints and gene expression are plotted with local motif z-scores and global footprint FDRs. (b) Local log2 fold changes for TNBC patient tumour footprints are plotted with local motif z-scores and global footprint FDRs and *TWEAK* gene expression fold changes. TF binding changes exhibiting p-values in the upper half of the distribution are selected and filtered for consistent global footprint and gene expression dynamics across cell lines and tumours. Motifs are clustered based on similarity via TOBIAS. (c) MAFG protein expression analysed by western blotting in MDA-MB-231, Hs578T, SKBR3, MDA-MB-453, MCF7, T47D and CAMA1 cell lines. (d) MAFG, p52, and H3K27ac ChIP-seq, and ATAC-seq signals of MDA-MB-231 cells treated with and without TWEAK at TWEAK/Fn14-driven TNBC differential H3K27ac peaks. (e) Correlation between *MAFG* and *NAMPT* expression in the TCGA BRCA and TCGA PanCancer RNA-seq dataset. (f) NAMPT locus. Top: MAFG ChIP-seq of untreated and TWEAK-treated MDA-MB-231, Hs578T, SKBR3 and MCF7 cells. Bottom: H3K27ac ChIP-

seq of untreated and TWEAK-treated MDA-MB-231 cells. The western blot samples derive from the same experiment and same gel for MAFG and GAPDH. Source data are provided as a Source Data file.

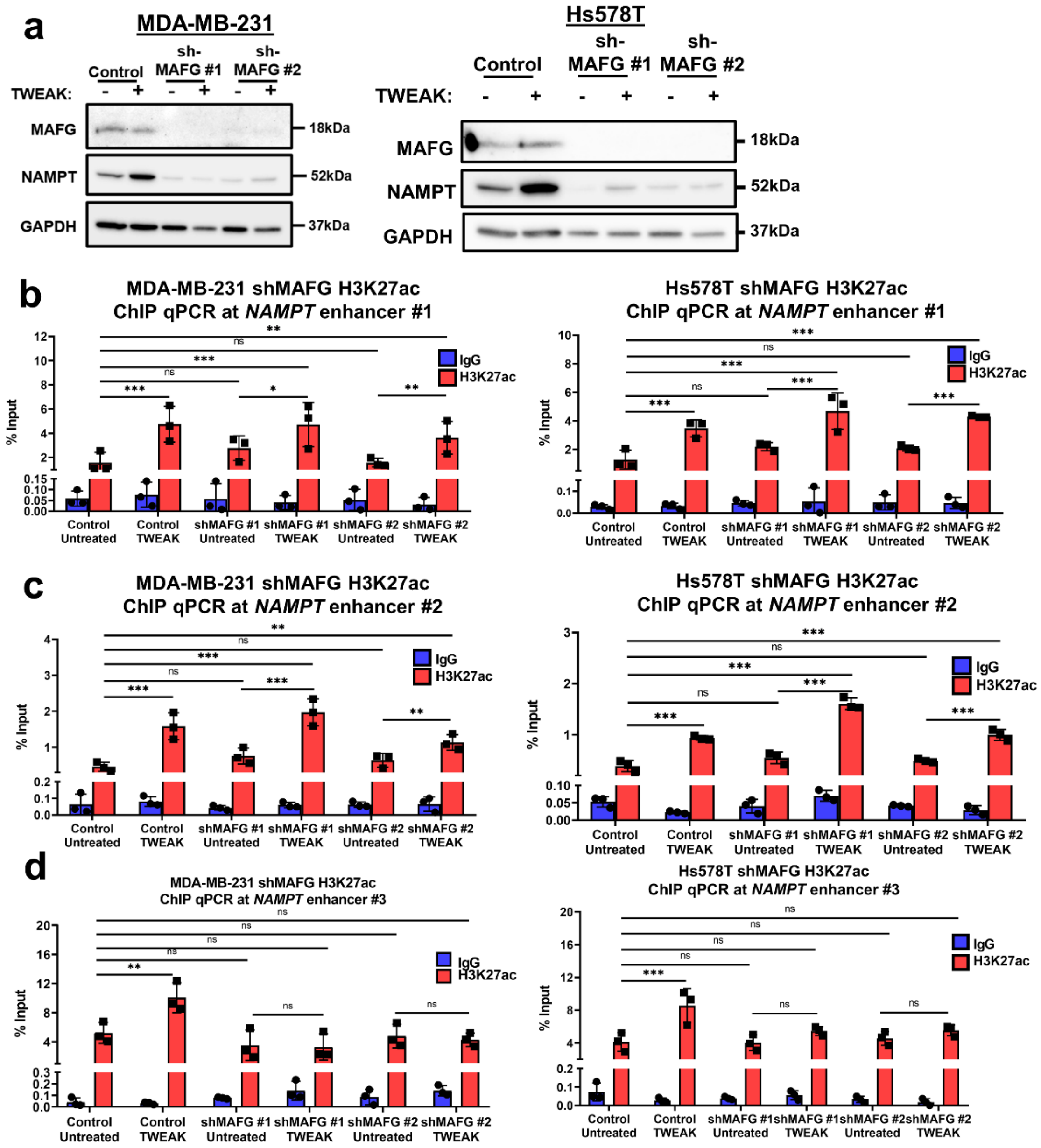

Supplementary Figure 17. MAFG is required for TWEAK/Fn14-driven NAMPT expression and *NAMPT* enhancer #3 activation. (a) MAFG and NAMPT protein expression analysed by western blotting in Control and shMAFG MDA-MB-231 and Hs578T cells treated with and without TWEAK. Plots depicting the IgG and H3K27ac ChIP qPCR %input at (b) *NAMPT* enhancer #1, (c) *NAMPT* enhancer #2 and (d) *NAMPT* enhancer #3 in control and shMAFG MDA-MB-231 and Hs578T cells treated with and without TWEAK (mean ± s.d). Data shown represent n=3 biological replicates. Experiments involving cell lines were performed 3 times independently, each time on different days. The western blot samples derive from the same experiment and the same gel for NAMPT, MAFG and GAPDH. Two-sided two-way ANOVA was used for statistical analysis where *P < 0.05; **P < 0.01; ***P < 0.001. Source data are provided as a Source Data file.

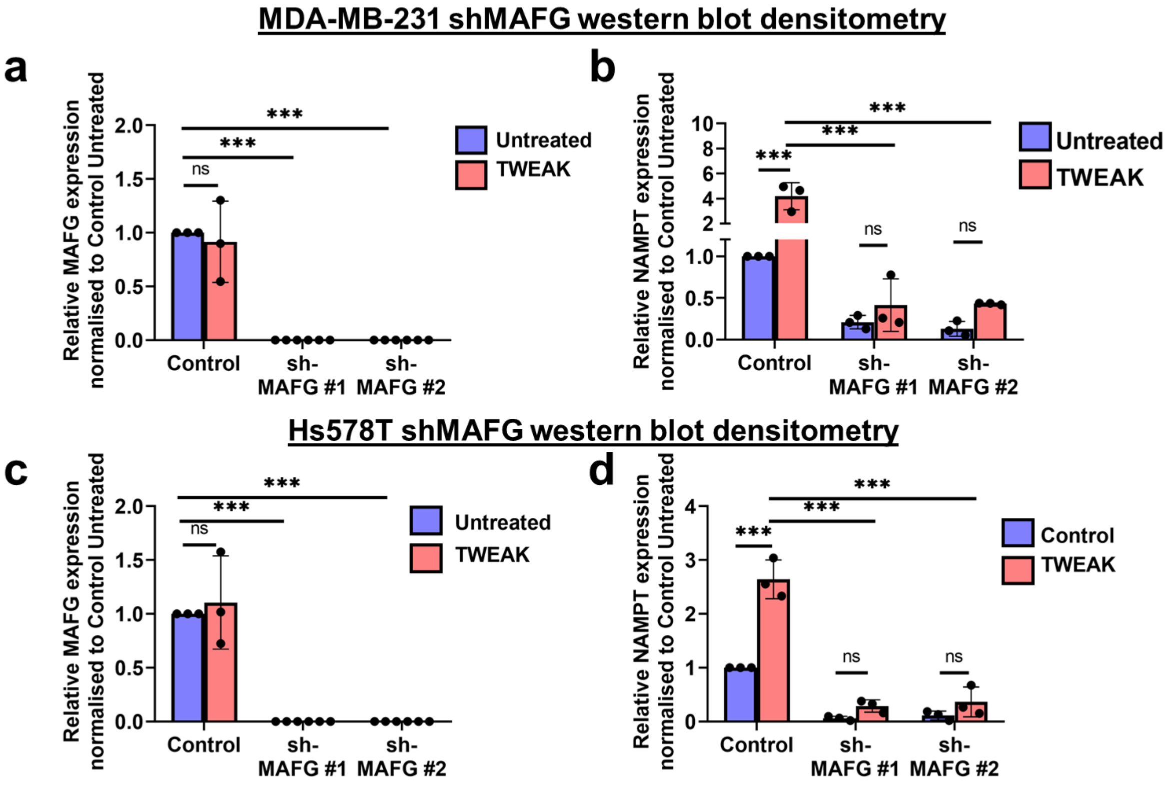

Supplementary Figure 18. Western blot densitometries. Relative (a) MAFG and (b) NAMPT expression to GAPDH, normalised to the control untreated MDA-MB-231 sample in Supplementary Figure 17a. Relative (a) MAFG and (b) NAMPT expression to GAPDH, normalised to the control untreated Hs578T sample in Supplementary Figure 17a. Two-sided two-way ANOVA was used for statistical analysis, where *P < 0.05; **P < 0.01; ***P < 0.001. Source data are provided as a Source Data file.

a

b

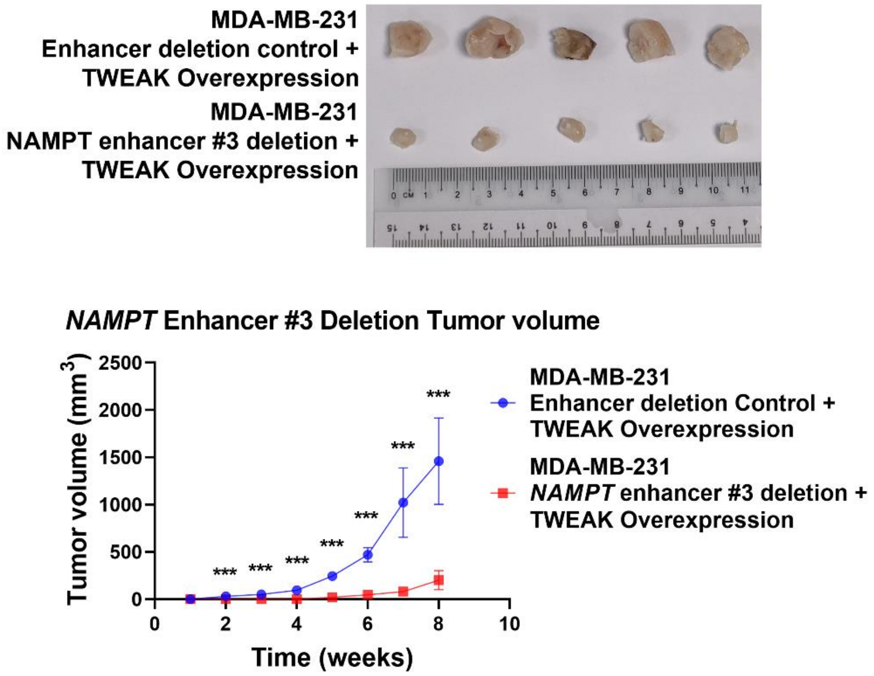

Supplementary Figure 19. *NAMPT* enhancer #3 deletion ameliorates TWEAK/Fn14-driven TNBC tumour growth. (a) Images of tumours extracted from mice 8 weeks after being injected with luciferase and TWEAK overexpressing MDA-MB-231 cells that either harbour the *NAMPT* enhancer #3 deletion or without (n=5 biological replicates). (b) Tumour growth plot depicts the weekly average tumour volume (mean ± s.d) from the mice. Performed in n=5 biological replicates. Two-sided t-test was used for statistical analysis where *P < 0.05; **P < 0.01; ***P < 0.001. Source data are provided as a Source Data file.

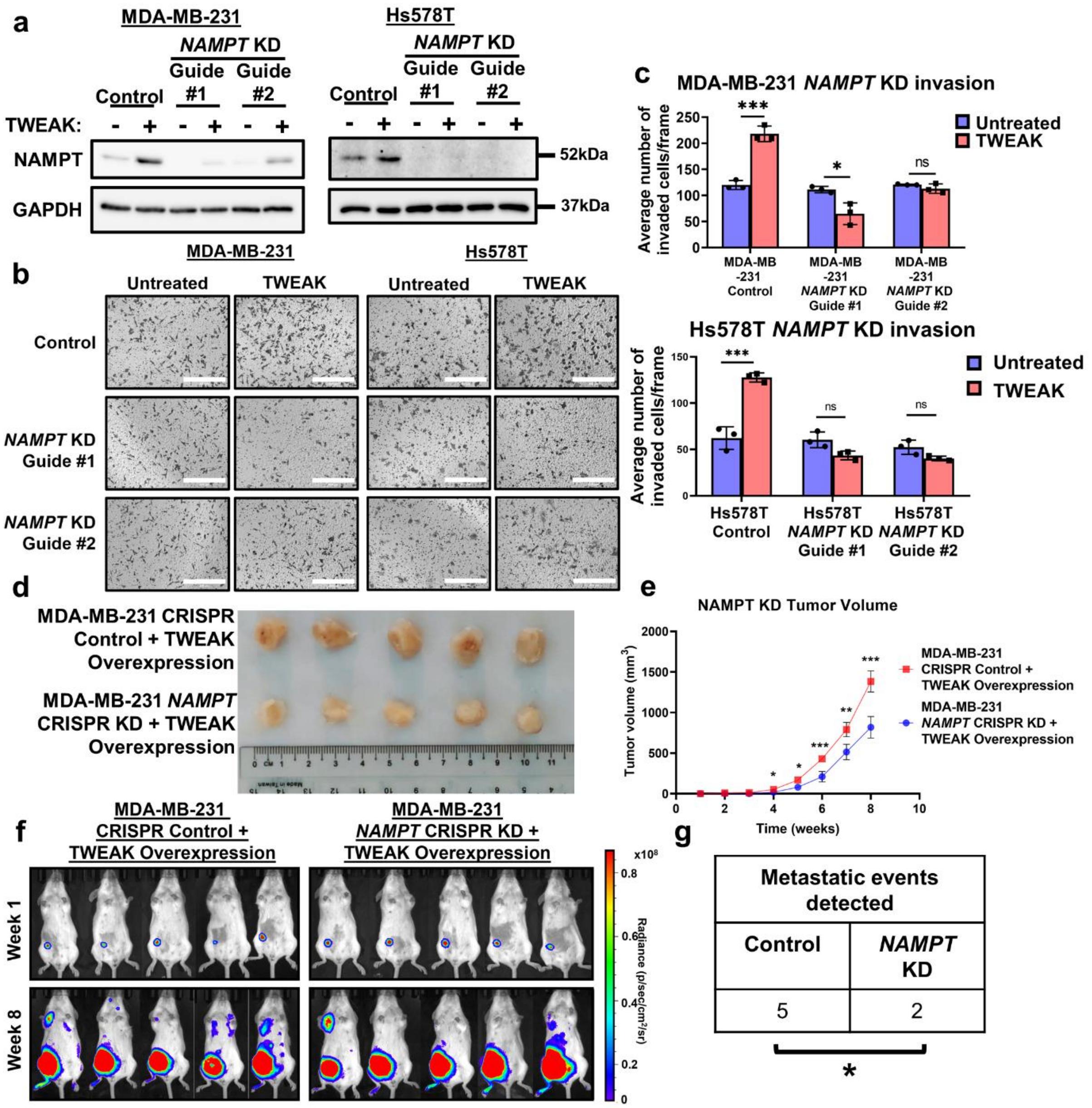

Supplementary Figure 20. NAMPT is a critical regulator of TWEAK/Fn14-driven tumour growth and metastasis in TNBC. (a) NAMPT expression analysed by western blotting in control and *NAMPT* KD MDA-MB-231 and Hs578T cells, with and without TWEAK treatment. (b) Transwell invasion assay was performed in control and *NAMPT* KD MDA-MB-231 and Hs578T cells, with and without TWEAK treatment. Representative images from n=3 biological replicates are shown. Scale bars: 400μm. (c) Representative plot depicts the average number of invaded cells/frame (mean ± s.d) from n=3 biological replicates, across four fields per replicate. (d) Images of tumours extracted from mice 8 weeks after being injected with luciferase and TWEAK overexpressing control and *NAMPT* KD MDA-MB-231 cells (n=5 biological replicates). (e) Tumour growth plot depicts the weekly average tumour volume (mean ± s.d). Performed in n=5 biological replicates. (f) IVIS tracking of mice injected with luciferase and TWEAK overexpressing control and *NAMPT* KD MDA-MB-231 cells. Representative bioluminescent images of the animals were taken at 1 and 8 weeks after orthotopic xenograft. (g) Plot depicts the number of metastatic events observed in animals after 42 days in n=5 biological replicates. Data presented as binomial. The western blot samples derive from the same experiment and same gel for NAMPT and GAPDH.

Experiments involving cell lines were performed 3 times independently, each time on different days. Two-sided two-way ANOVA was used for statistical analysis in *in vitro* invasion assay. Two-sided t-test was used for statistical analysis in *in vivo* assays. *P < 0.05; **P < 0.01; ***P < 0.001. Source data are provided as a Source Data file.

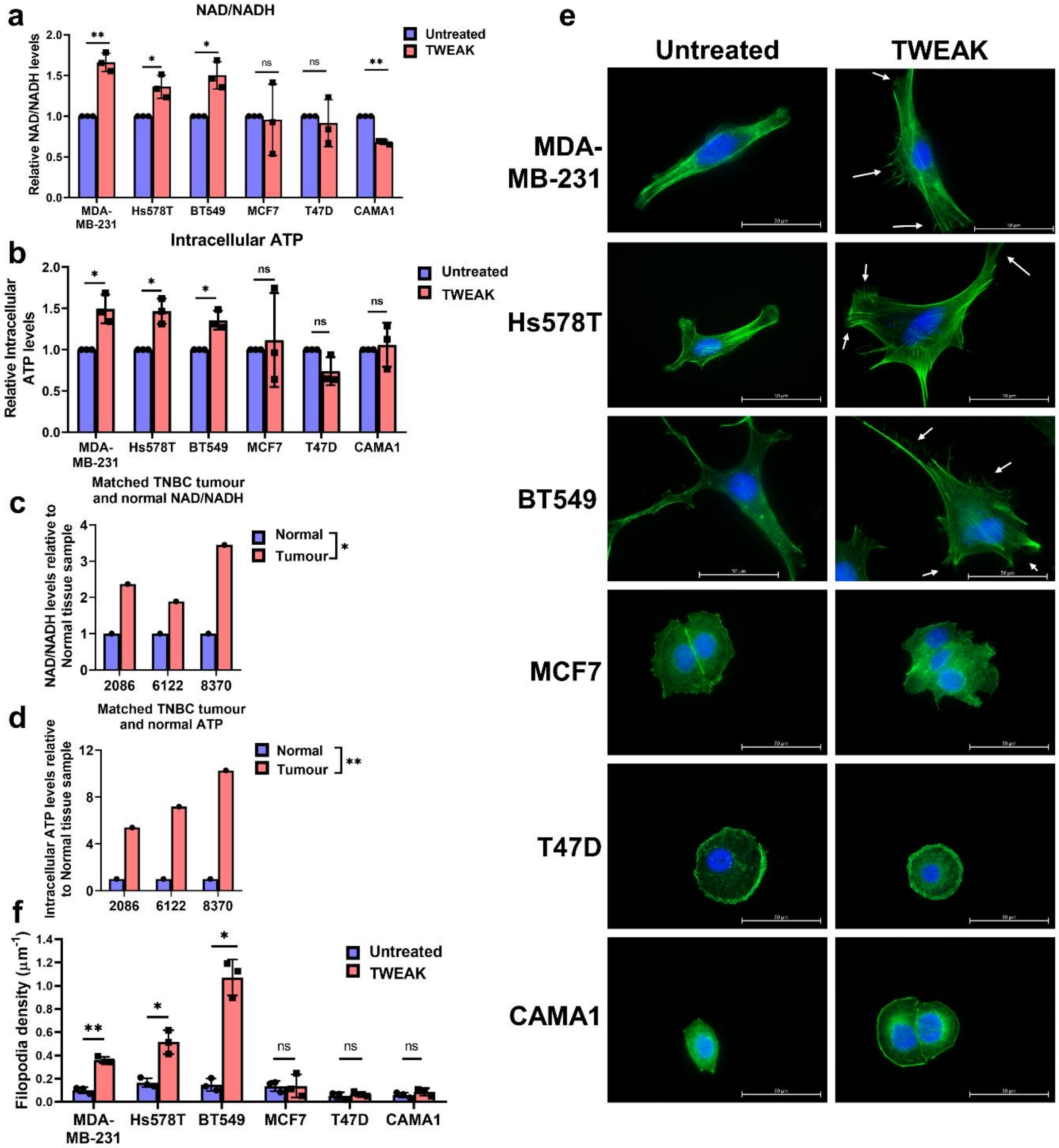

Supplementary Figure 21. TWEAK/Fn14 activation promotes NAD+/NADH and intracellular ATP production and stimulates filopodia formation in TNBC cell lines. Bar plot depicting the relative (a) NAD+/NADH and (b) intracellular ATP levels in MDA-MB-231, Hs578T, BT549, MCF7, T47D and CAMA1 cells, with and without TWEAK. (c) NAD+/NADH and (d) intracellular ATP levels in Fn14 and NAMPT high TNBC tumours and their matched normal samples (2086, 6122 and 8370). (e) Representative images of MDA-MB-231, Hs578T, BT549, MCF7, T47D and CAMA1 cells stained for actin and DAPI, with and without TWEAK treatment, using a Zeiss Live Cell Observer microscope (63x magnification). White arrows point to filopodia protrusions. Scale bars: 50µm. (n=3 biological replicates) (f) Plot depicting

the average filopodia density (mean ± s.d) from n=3 biological replicates. Experiments involving cell lines were performed 3 times independently, each time on different days. Two-sided t-test was used for statistical analysis in all assays. *P < 0.05; **P < 0.01; ***P < 0.001. Source data are provided as a Source Data file.

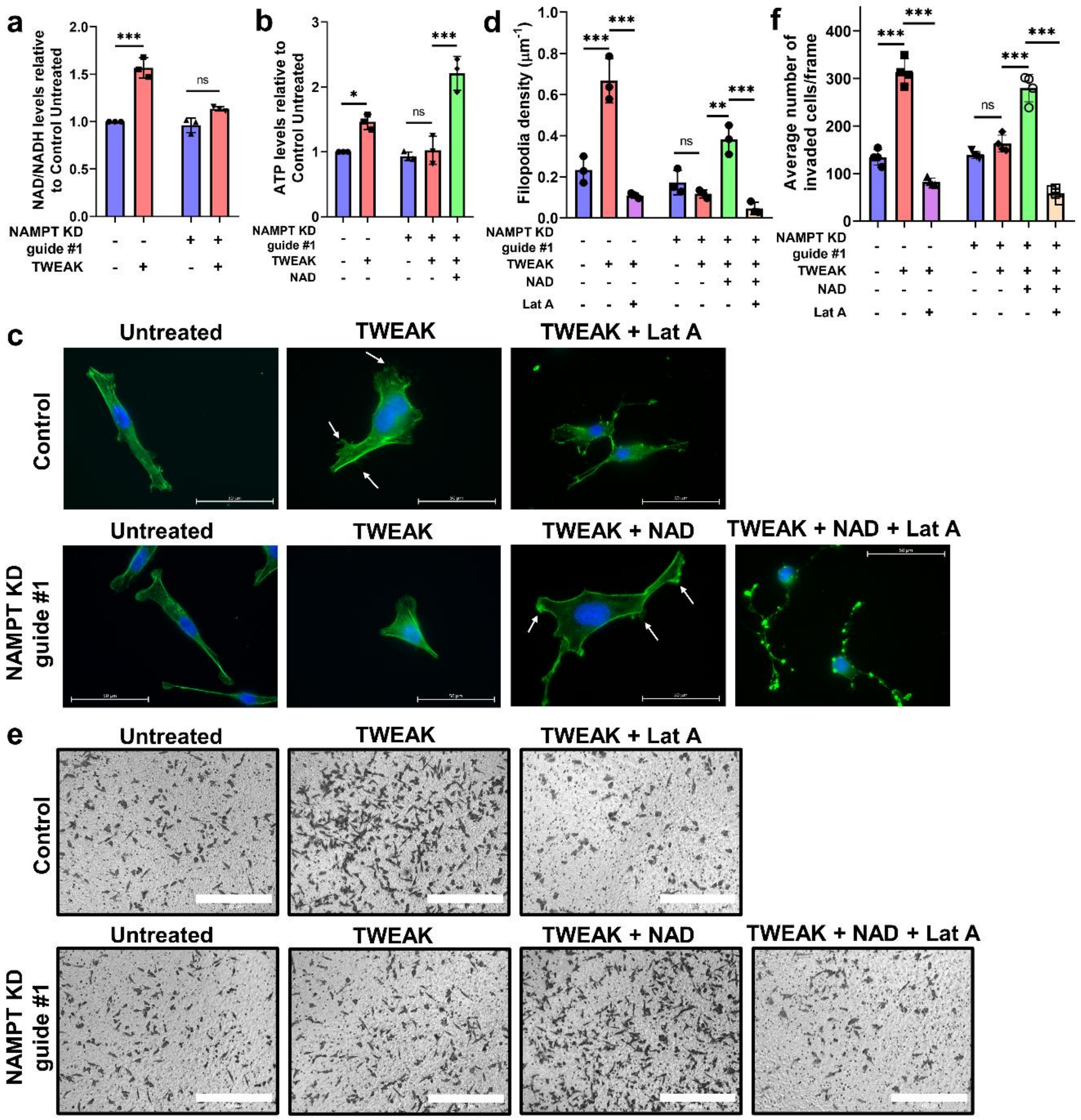

Supplementary Figure 22. NAMPT is important for TWEAK/Fn14-driven filopodia formation and invasion of TNBC cells. (a) Plot depicting the relative NAD+/NADH levels in control and *NAMPT* KD MDA-MB-231 cells, with and without TWEAK treatment. (b) Plot depicting the relative intracellular ATP levels in control and *NAMPT* KD MDA-MB-231 cells, following TWEAK and NAD+ treatment. (c) Representative images of control and *NAMPT* KD MDA-MB-231 cells stained for actin and DAPI, following TWEAK, NAD+ and LatA treatment, using a Zeiss Live Cell Observer microscope (63x magnification). White arrows point to filopodia protrusions. Scale bars: 50μm. (n=3 biological replicates) (d) Plot depicting the average filopodia density (mean ± s.d) from n=3 biological replicates. (e) Representative images of transwell invasion assay performed in Control and *NAMPT* KD MDA-MB-231 cells

following TWEAK, NAD+ and LatA treatment (n=3 biological replicates). Scale bars: 400μm. (f) Plot depicts the average number of invaded cells/frame (mean ± s.d) from n=3 biological replicates, across four fields per replicate. Experiments involving cell lines were performed 3 times independently, each time on different days. Two-sided one-way ANOVA was used for statistical analysis in all assays. *P < 0.05; **P < 0.01; ***P < 0.001. Source data are provided as a Source Data file.

Name	Sequence
Fn14 KO Guide #1	AGAAAGCAGCCCCAGCACGA
Fn14 KO Guide #2	TCAGGCTCAGAGCGCCCCCA
NAMPT KD Guide #1	ATTAAGGAAGGTGAAATATG
NAMPT KD Guide #2	CTGGGAATGACAAAGCCCTC
NAMPT enhancer deletion #1 upstream Guide	GTGGGGATGGTATAAACACC
NAMPT enhancer deletion #1 downstream Guide	TAAGGTGGAAGTACAGACAC
NAMPT enhancer deletion #2 upstream Guide	GCAGGAAATGAGGGACAACG
NAMPT enhancer deletion #2 downstream Guide	ATCATCAAAACAGAGCCAGT
NAMPT enhancer deletion #3 upstream Guide	AAGTGCTCTCAGAGTCCATG
NAMPT enhancer deletion #3 downstream Guide	GACATGTTTGATAACCCAATG
Mouse Fn14 guide	CTTGGTGTTGATGCGCGCCG

Supplementary Table 1. sgRNA guide sequences used for CRISPR plasmids.

Name	Sequence
shMAFG #1	CCAGCGTCATCACAATAGTAA
shMAFG #2	CCTCAGAGAACGCCAGCATGA

Supplementary Table 2: shRNA sequences used.

Name	Sequence
TWEAK F	ATACCGGTCGCCACCATGAAAACACGGGCTCGAAG
TWEAK R	CGTATTCGAATCAGTGAACCTGGAAGAGTCCGAAGTAGGT

Supplementary Table 3: Primer sequences used to amplify sTWEAK.

Name	Sequence
NAMPT enhancer #1 F	AAAGATAGGGTGGGAGAAGGAAAC
NAMPT enhancer #1 R	CAGGAAAATCACATGGAGCTTTGT
NAMPT enhancer #2 F	CCAGAATAAGAGTTCCCTTGGGAT
NAMPT enhancer #2 R	AAGGAGCTTCTGAAGATATTCCCC
NAMPT enhancer #3 F	TTCCACTGACATAGGCAATACTCC
NAMPT enhancer #3 R	TTCTGTGTCCTTTGTTCCAATCAC
Fn14 KO F	GAAATCATTCGGGAGGAGGTGGGAG
Fn14 KO R	GGATGAATGAATGATGAGTGGGCGA

Supplementary Table 4: Sequencing primers used for genotyping CRISPR clones.

Name	Sequence
NAMPT enhancer #1 F	ACCGCTGTTTATTGGGCATTT
NAMPT enhancer #1 R	CCCACTCCACTGCTTGCTAG
NAMPT enhancer #2 F	CCCCACTGGCTCTGTTTTGA
NAMPT enhancer #2 R	CACTCAACCCCTTCTCCCAC
NAMPT enhancer #3 F	AGTGCTTCCCTTCCCTGAGA
NAMPT enhancer #3 R	GCGGAAGTAAGCATCGTGAC

Supplementary Table 5: ChIP qPCR primers.

References

1. Xu K, Wang R, Xie H, Hu L, Wang C, Xu J, et al. Single-cell RNA sequencing reveals cell heterogeneity and transcriptome profile of breast cancer lymph node metastasis. Oncogenesis. 2021;10(10):66.
